# Nasopharyngeal Tonsils (Adenoids) Contain Extrathymic Corticothymocytes

**DOI:** 10.1371/journal.pone.0098222

**Published:** 2014-05-23

**Authors:** Serena Buscone, Werner Garavello, Fabio Pagni, Renato Maria Gaini, Giorgio Cattoretti

**Affiliations:** 1 Anatomic Pathology, Department of Surgery and Translational Medicine, Universitá degli Studi di Milano-Bicocca, Milano, Italy; 2 Otolaryngology, Department of Surgery and Translational Medicine, Universitá degli Studi di Milano-Bicocca, Milano, Italy; 3 Pathology, Azienda Ospedaliera San Gerardo, Monza, Italy; INRS - Institut Armand Frappier, Canada

## Abstract

Adenoidal tissue (also known as nasopharyngeal tonsils) of 58% of humans in the pediatric age group contains immature T-lymphoid cells with the phenotype of thymocytes (TdT+,CD1abc+, cytoplasmic CD3+, coexpressing CD4 and CD8, lacking an Intraepithelial Lymphocyte-associated phenotype). The notable difference in comparison to palatine tonsils is the clustering in groups and sheets, comprising hundreds or thousands of cells (1.7%±0.2 of total T cells). The thymic epithelium is morphologically and phenotypically absent. Adenoids share with tonsils and lymph nodes the presence of immature B cell precursors (TdT+, CD1a-, Pax5+, Surrogate light chain±), however in these latter the presence of TdT+, CD1a+, Pax5- precursors is absent or limited to individual cells. Human adenoids are distinct among the Waldeyer's ring lymphoid tissue because of the known embryogenic derivation from the third pharyngeal pouch, from which the thymus develops; in addition, they may display phenotypic incomplete features of a vestigial thymus.

## Introduction

T- and B-cell lymphoid precursors, named after two lymphopoietic organs (the thymus and the bursa, the chicken equivalent of the human adult bone marrow) are characterized by unique and shared components of a variegated repertoire of proteins, among which are T- and B-cell receptors, DNA recombinases (RAG-1 and RAG-2) targeting the genes coding for those receptors and other enzymes accessory to the recombination complex such as Terminal Deoxynucleotidyl Transferase (TdT).

The B-cell development in the bone marrow has been elucidated to great extent with liquid- and single-cell suspension based methods as well as cell culture and transplantation experiments, but the tissue organization of the B cell development is less detailed, compared to the thymus.

In the thymus, an epithelial organ acts as niche and contributes to the expansion, differentiation and immunological education of thymocytes [1]. Its unique commixture of epithelial and immature T-lymphoid cells has facilitated the study of fully committed T cells.

Studies on athymic mice have identified extrathymic T cell progenitors in the gut and mesenteric lymph nodes which are able to differentiate into mature T cell subsets [2], [3]. This has been confirmed in humans, at least in the gut [4] and in the bone marrow [5], while the characterization of the extrathymic immigrants has remained somewhat elusive [6].

In the mouse there is abundant evidence of extrathymic T-lymphopoiesis [2], [3] but also a fully functional, anatomically complete, extrathoracic second thymus [7], [8]. This latter depends on thymic stroma-inducing factors absent in other lymphoid tissue locations, driven by genes whose human orthologs are located at the chromosome 22q11.2 locus.

Extrathymic human T-lymphopoiesis has been demonstrated in the palatine tonsil [9] and the gut [4], along abundant evidence of extramedullary B-lymphopoiesis [10], [11], [12], [13].

In these instances, in situ demonstration of immature lymphoid cells has been complemented with ex-vivo cell culture or extractive biochemical methods, showing the ability of the putative precursors to further differentiate and to display a coordinated immature phenotype. Rarely, if ever, these extrathymic precursors amounted to more than scattered, isolated cells in the tissue, compared e.g. to the thymus.

In both bone marrow and thymus, TdT is a central marker to position a lymphoid cell along its maturation path in the tissue [14], [15].

Indolent proliferations of benign T-lymphoblasts have been described in various conditions and body sites [16], [17], [18], [19], [20]. These proliferations often involve the oropharynx [19], [20], the salivary glands [16] and tissues known to harbor hemolymphopoiesis during fetal development (liver, mesentery) [18], [21].

Since oropharyngeal mucosa-associated lymphoid tissue (MALT) (palatine, lingual, nasopharyngeal tonsils [also known as adenoids] and Waldeyer's ring-associated lymphoid tissue) has diverse origin during embryonic development, we decided to investigate palatine and adenoidal tissue separately, in specimens excised in the pediatric age group.

## Materials and Methods

All human tissues were leftover normal tissue, anonymous, obtained from diagnostic or curative surgical procedures. The majority of the samples have been obtained up to 2006 at the Columbia Presbyterian Medical Center, USA, and covered by permission or exempt by the Columbia University Institutional Review Board [22] and/or exempt from informed consent being residual material after diagnosis and fully anonymized, as per San Gerardo Hospital regulations released by the Ethics Committee (Comitato Etico Indipendente, ASG-IA-017 Donazione di materiale biologico a scopo di ricerca e_o sperimentazione, May 2012). Lymphoid tissue was collected and analyzed as previously described [22], [23] and consisted of 17 palatine tonsil and adenoids from the same patient, 25 uninvolved lymph nodes, mostly axillary, from 25 cancer staging procedures and seven spleens removed for non-hematological diseases. Lymph nodes with follicular hyperplasia were not included. None of the tonsil and adenoids showed abnormality in macroscopic aspect, unilateral growth or pathologic enlargement, both macroscopic or microscopic. Specimens with any history of concurrent or past pathology were discarded, with the only exception of ear and nose trivial ailments, typical of the pediatric age.

The primary antibodies used for immunohistochemistry/immunofluorescence were rabbit anti-TdT (Supertechs, Bethesda, MD, 1∶200), pooled anti human λ5 (HSL11) and VpreB (HSL96) IgG1 MoAbs [24] at 1 µg/ml each (frozen tissue only), CD3ε, CD5, CD7, CD20, CD68R, CD79a, rabbit anti myeloperoxidase, anti-S100, TP53, bcl2 (DAKO, Glostrup, Dk), CD1a, CD10, CD30, CD34, (Novocastra Laboratories, Newcastle-U-Tyne, UK), CD3ζ, PU.1, IRF4, IRF7, IRF8, (Santa Cruz Biotechnology, Santa Cruz, CA), Pax-5, rabbit anti CD19 (Transduction Laboratories, San Diego, CA), CD56, anti-keratin, negative control ascites (Sigma-Aldrich, Milan, Italy), CD10 (clone PHM6) (Serotec, Raleigh, NC),, CD19, CD10, CD103, CD22, GranzymeB (Coulter/Immunotech, Fullerton, CA), LEF-1/RMB6, p75-NGFR (ThermoFisher, Raleigh, CA). For flow cytometry, the antibodies used were CD38-Pe, CD4-FITC, CD8-Pe, CD4-Pe (Pharmingen/BD Biosciences), CD3Pe/CD8FITC (Coulter/Immunotech).

CD1a, CD1b, CD1c, anti-thymic epithelium antibodies (T905/RFD4, T895/TE4-2G10, TE-7) were from the 2^nd^ and 3^rd^ International Workshop on Human Leukocyte Differentiation Antigens [25]. Anti TCR-β antibody βF1 was a gift from MB. Brenner (Harvard Medical School, Boston, MA). Ki-67 MIB 1 antibody was from J. Gerdes (Molecular Immunology, Borstel, FRG). FoxN1 staining was not investigated because negativity in postnatal thymus may not exclude the presence of thymic epithelium [26].

Mononuclear cell suspensions, stained in direct and indirect four color immunofluorescence with directly conjugated primary antibodies as previously described [22], [23], were analyzed in a Becton Dickinson (Sunnyvale, CA) Excalibur flow cytometer and analyzed with the FlowJo software (Tree Star, Inc., San Carlos CA.).

In selected cases, we used density- or magnetic-enriched tonsil and adenoid T-cell samples from unrelated experiments aimed at B-cell characterization [22], [27].

TdT-mediated end-labeling of fragmented DNA (TUNEL) in apoptotic nuclei was performed as described by the supplier (Roche, Monza, Italy) [27].

### Imaging

Gray scale or color images were taken on an E600-Nikon Microscope (NikonUSA, Melville, NY), fitted with Planachromat 4x/0.10/30.0, 10x/0.25/10.5, 20x/0.40/1.3, PlanApo 40x/.095/.12-.16 (light microscopy) or PlanFluor 10x/0.30/16.0, 40x/0.75/0.72, 60x/0.80/0.3 (immunofluorescence) objectives, with a SPOT-2 CCD camera and software (Diagnostic Instruments Incorporated, Sterling Heights, MI).

All images were edited for optimal color contrast with Adobe Photoshop7 and Adobe Illustrator 10 (Adobe Systems Incorporated, San Jose, CA), on a G4 Apple computer (Apple Computers, Cupertino, CA).

### Morphometry

Five µm tissue sections were scored with a microscope grid. Measurements of number of nuclear-stained cells per square mm were obtained from the total tissue section surface, or the area encompassing the medullary cords, the non-paracortical and the non-follicular zones in LNs, the area between the septal fibrous tissue or the subepithelium and the follicular marginal zone in tonsils, where TdT+ cells gather.

## Results

Adenoids (also known as nasopharyngeal tonsils [28]) contain, besides abundant mature peripheral lymphoid tissue, immature lymphoid cells with the characteristic of cortical thymocytes: TdT+, CD1_abc_+, cytoplasmic CD3εζ+, CD4+, CD8+, CD10±. All adenoids examined contained immature T cells but the amount was very heterogeneously distributed and variable among individuals. The complete phenotype of the TdT+ cells obtained on at least three samples, both on frozen and fixed and embedded sections was: Pax-5-, CD1_abc_+, CD2+, surfaceCD3ε-, cytoplasmic CD3εζ+, CD4+, CD5+, CD7+, CD8+, CD10±, bcl2-, CD19-, CD20-, CD22-, CD30-, CD38+, CD56-, CD79a-, CD103-, Surrogate Light Chain (SL)-, TCRβ+, Granzyme B-, TP53-, PU.1-, IRF4-, IRF7-, IRF8-, LEF-1+, CD68R-, S-100-, MPO-, Ki-67+.

**Figure 1:**
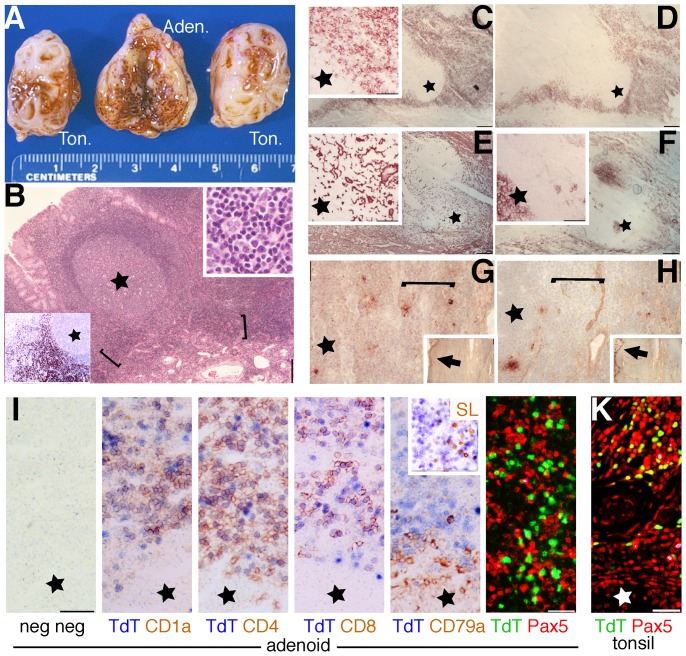
Phenotype and distribution of immature T cells in adenoids and tonsils. A: macroscopic appearance of a representative sample of tonsils and adenoidal tissue. B: Low power (4x) image of a fixed and embedded adenoid, stained with Hematoxylin and Eosin. The brackets highlights the boundary between the stroma and the secondary lymphoid tissue (star  =  germinal center) where darker staining small lymphocytes gather. Left lower corner inset shows TdT+(brown) cells in sheets in an immunostained serial section (4x). Upper right inset: H&E detail of the bracketed area (40x). C-H: frozen adenoid serial sections (4x) stained for CD1a (C), CD1b (D), TE-7 (E), NGFR/p75 (F), and the anti thymic epithelium reagents RFD4 (G) and TE-4 (H) (brown, no counterstain). The insets in C, E and F show a magnified detail of the area occupied by immature T cells (40x)(also indicated by brackets). Star  =  germinal center. The insets in G and H show low power images (4x). The arrows highlight positive surface epithelium. I: frozen adenoid serial sections (40x) double stained for negative controls, TdT and CD1a, CD4, CD8, CD79a and surrogate light chain (SL; inset). Note the doublestaining in the upper half field, except for CD79a and SL. The last image is a double immunofluorescence image showing largely mutually exclusive distribution of Pax5 and TdT in adenoids in a fixed and embedded section. K: fixed and embedded tonsil section showing coexpression of TdT and Pax5 in immature B cell precursors.

**Figure 2:**
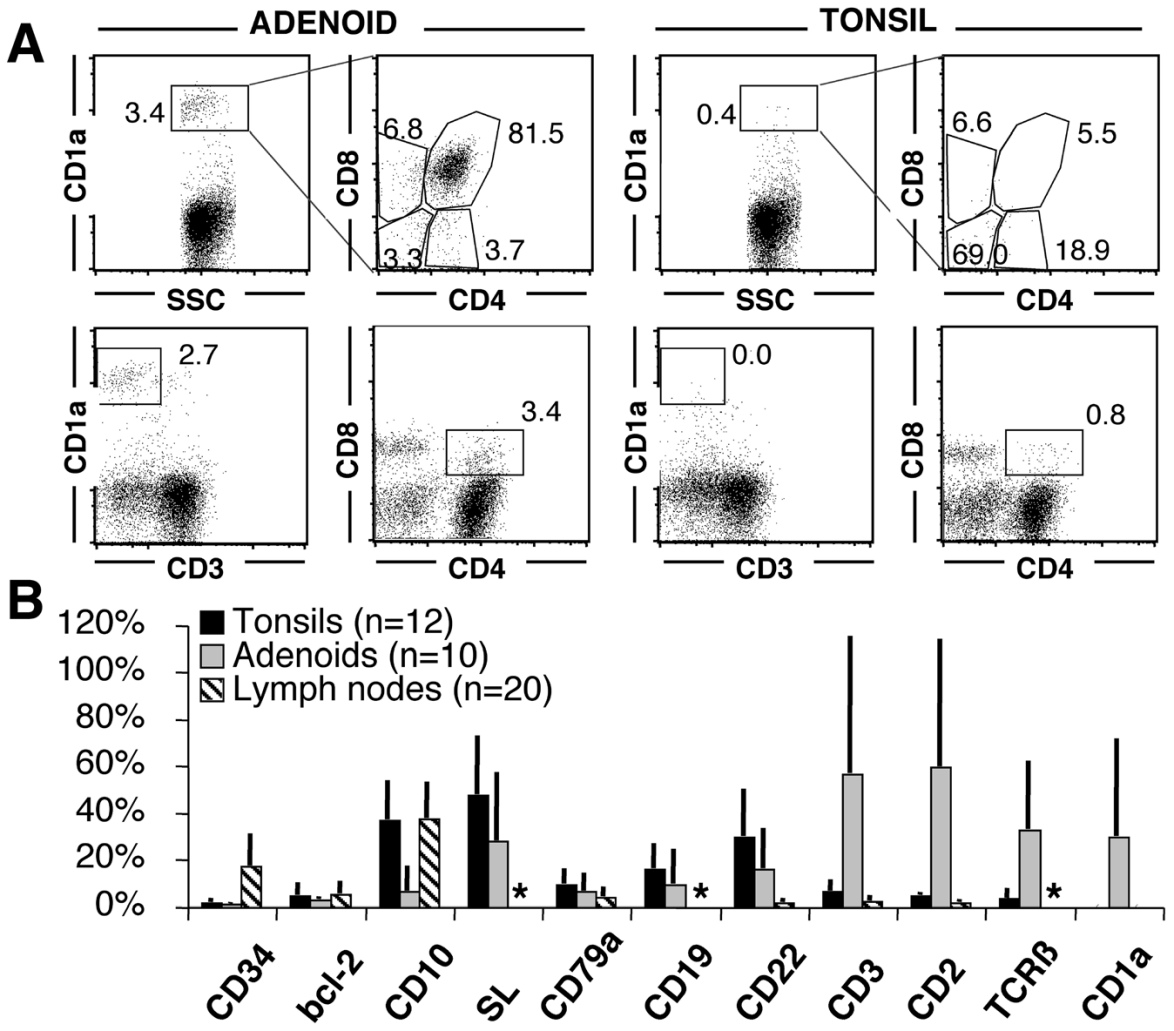
Phenotype of TdT+ cells in adenoids, tonsils and secondary lymphoid organs. A: Adenoid and tonsil from the same individual, stained by flow cytometry for surface antigens, show in the adenoid only CD1a+, surface CD3-, double CD4 and CD8 positive immature T cells. The tonsil is devoid of such cells. The numbers indicate the percentage in each gate. CD2+ T cells were enriched by SRBC rosetting. B: The phenotype of TdT+ cells in 12 tonsils, 10 adenoids and 20 lymph nodes is plotted. Each marker has been counted on TdT+ cells and the mean ± SD is shown. The variability is due to dilution with TdT+ immature B cells. An asterisk marks analysis not done. Note the unique presence of CD1a+ immature T cells in adenoids.

CD1_a_+ TdT+ cell expansions (defined as TdT+ clusters identifiable at 4x) were unique to adenoids (10 out of 17, 58.8%) (Figure 1) and not found by immunohistochemistry in 25 lymph nodes, 6 additional palatine tonsils or 7 spleens (Figure 2). By flow cytometry, 1.7% ±0.2 (n = 4) adenoidal cells were brightly CD1_a_+, coexpressing CD4 (40.1%±12.9), CD8 (87.5%±8.0) and dimly CD10 (34.0%±2.1). The TdT+ elements are small lymphoblasts/lymphocytes, located in the interfollicular spaces, at the base of the reactive B cell follicles, as dense homogeneous sheets. Phagocyting macrophages ingesting apoptotic cells were not identified and TUNEL+ cells in these areas did not differ in frequency from other interfollicular areas devoid of TdT+ elements (pos areas: 1.13±0.6 TUNEL+/sqmm; neg areas: 1.28±0.35 TUNEL+/sqmm; n = 4; p = 0.35). No clonal products of the T-cell receptor locus were identified in a case with prominent lymphoblasts presence (not shown).

As described in palatine tonsils [10] and lymph nodes [13], immature B cell precursors (TdT+, Pax5+, CD10+, SL+, CD79a+, CD22+, CD3-) are found in small amounts adjacent to the thymic cell expansion. Very rare isolated TdT+ CD3+ precursors were identified in tonsils and lymph nodes. The rare CD34+ lymphoid cells we did found in all organs could not reliably be assigned to a B- or T-cell lineage commitment.

No morphologically or phenotypically recognizable thymic epithelial tissue can be identified in areas of lymphoblasts expansion, thus differentiating adenoidal thymopoiesis from ectopic cervical thymus. TE7+, p75/NGFR-, RFD4-, TE4-2G10- cytokeratin 1, 5, 10, 14 (Ab 34βE12) negative fibroblasts populated these areas.

## Discussion

Nasopharyngeal tonsils, or, as commonly referred to, adenoids are an additional, previously unreported site where human immature lymphoid cells with a thymic phenotype are found, with a distinctive difference compared to other oropharyngeal [28] and extrathymic lymphoid tissue [4]: the focal gathering in large numbers, albeit in some subjects.

The absence of a phenotypically recognizable epithelial stroma indicate that a yet unidentified stromal cell type is permissive for thymocyte homing or developing in the human adenoidal tissue. Immature B-lymphopoiesis may require a more promiscuous or ubiquitous milieu, since they can be observed in the tonsils, adenoids, lymph nodes, spleen [10], [11], [13] and even thymus [29].

An increased number of CD1a+ mononuclear cells, up to one log higher than in tonsils, was reported by Fokkens et al. [30]. Papatziamos et al. [31] similarly described focal gathering of CD1a+ lymphoid cells in two out of fifteen adenoids. In neither case the nature of these cells was further investigated, nor the findings have been deemed sufficient to separate the adenoids from he rest of the Waldeyer's ring lymphoid tissue (besides the anatomy).

The absence of a consistent CD103+, CD56+, TCRγ and CD8 phenotype [32], [33] suggests that adenoidal immature T cells are different from the described phenotype of intraepithelial lymphocyte and closer if not identical to thymus cells. It is also unlikely that these cells represents mature T cells undergoing receptor revision and re-expression of TdT, RAG-1 and RAG-2 [34] since they have an immature phenotype and express TCRβ, at variance with the receptor editing mature T cells [34].

The absence of clonal rearrangements of the T-cell receptor, even when these cells expand to a surgically resectable size [17], rules out a malignant or pre-malignant condition. On the other side, quantification of T-cell receptor rearrangement excision circles (TRECS) [35], a molecular sign of ongoing TCR rearrangement, in two tonsils and four adenoids, half of each containing detectable TdT+ elements did not show significant differences because of extremely low quantities (not shown) and dilution by tissue-resident and circulating TREC-bearing peripheral blood cells, increased in the pediatric age range [36]. Thus, a direct proof of in situ maturation is still elusive.

Our findings may imply extrathymic thymopoiesis or selective accumulation of circulating thymocytes.

CD34+ precursors may recirculate and seed a permissive microenvironment for lymphoid differentiation [9], which sustains local expansion and full thymocyte differentiation only in adenoids. McClory et al. [9] described to great detail the full range of immature T cell differentiation in tonsils. We replicated their findings but did found exceedingly rare, single isolated TdT+ CD3+ precursors in palatine tonsils and lymph nodes, and never the expansions which we did observed in the adenoids. It is possible that rare CD34+ precursors are able to survive and execute a programmed thymic differentiation in any lymphoid tissue. By sophisticated flow cytometry and culture methods, these stochastic isolated events may be interpreted as locally relevant. Another possibility is that adenoidal tissue were inadvertently admixed with the palatine tonsils in the experiments, something that quite often happens with this humble tissue of no diagnostic relevance.

Notably, absence or severe hypoplasia of both adenoids [37] and thymus are seen in the VCF/DiGeorge syndrome or CATCH22 phenotype (OMIM #188400 and #192430), a complex of congenital defects of the third pharyngeal pouch development with variegated penetrance and severity.

Mouse models of the syndrome show that genes influencing the mouse orthologs of the genes commonly deleted in the human region 22q11.2, must be coordinately co-regulated [38], in order to allow correct morphogenesis of the third pharyngeal pouch derivatives and induction of the mesenchymal-epithelial transition in the thymus. The incomplete and strain dependent penetrance of the murine cervical thymus [8], [39] analogous to our findings in human adenoid, may be evidence that, further along the evolution of mammals, the ability to induce a cervical thymic epithelium and to sustain local thymic differentiation, vary among individuals and, as suggested by our human findings, the two may become dissociated.

If our findings imply local thymopoiesis, then the human adenoid may represent a vestigial second thymus with distinctive features: incomplete penetrance, absence of a thymic epithelium, absent evidence of negative selection such as apoptosis and phagocytosis, and lack of a distinct cortical and medullary zones. The identification and isolation of both T-lymphoid precursors and distinctive stromal cells in adenoids will open new field of knowledge in lymphoid cell biology. The Waldeyer's ring is open to immunomodulatory inhalants (antigens, pathogens) which are crucial to the buildup of an effective immune response or, if deranged, autoimmune diseases. It is also readily approached by inhalation therapy. This newly discovered thymocyte population may further elucidate T-lymphocyte extrathymic differentiation and open new field of therapeutic intervention. Lastly, adenoids should be investigated in human cases of T-cell deficiency, acquired or congenital [40], give the fact that extrathymic T-lymphopoiesis may be boosted in such cases [2].

We cannot exclude that the selective gathering of thymocytes in the adenoids is due to accumulation from recirculating thymocytes, something which has been observed in the peripheral blood in Adenosine Deaminase-deficient, treated children [41]. None of the subjects we analyzed had any disease except frequent common infections, yet a selective role of the adenoids in collecting thymocytes further highlights a vestigial thymic function of this organ.
